# Association of Sodium–Glucose Cotransporter 2 Inhibitor Use With Outcomes in Patients Undergoing Transcatheter Aortic Valve Implantation: A Systematic Review and GRADE‐Assessed Meta‐Analysis

**DOI:** 10.1155/cdr/8933859

**Published:** 2026-10-05

**Authors:** Ahmed Adel Mohamed, Batool Husain Husain, Tammam Khaled Dali, Mostafa Elsayed Ahmed, Ghaida Al Shawawreh, Ali Samir Galal, Mahmoud Abdelwahab Moussa, Abdallah M. Hibysh, Abdalrahman Salah Shehata, Youssef Ramadan, Osama M. Aldeeb, Tasneem Zanati Saeed, Mohammed Dibas

**Affiliations:** ^1^ Faculty of Medicine Zagazig University Zagazig Egypt zu.edu.eg; ^2^ Faculty of Medicine Cairo University Cairo Egypt cu.edu.eg; ^3^ Faculty of Medicine Mansoura University Al Mansurah Egypt mans.edu.eg; ^4^ Faculty of Medicine Assiut University Assiut Egypt aun.edu.eg; ^5^ Faculty of Medicine New Ismaila National University Ismaila Egypt; ^6^ Faculty of Medicine South Valley University Qena Egypt svu.edu.eg; ^7^ Faculty of Medicine October 6 University Giza Egypt o6u.edu.eg; ^8^ School of Medicine The University of Jordan Amman Jordan ju.edu.jo; ^9^ Department of Medicine Faculty of Medicine and Health Sciences An-Najah National University Nablus State of Palestine najah.edu

**Keywords:** aortic stenosis, dapagliflozin, heart failure, SGLT2 inhibitors, transcatheter aortic valve implantation

## Abstract

**Background:**

Patients undergoing transcatheter aortic valve implantation (TAVI) remain at risk of adverse cardiovascular and renal outcomes despite procedural advancements. Sodium–glucose cotransporter 2 (SGLT2) inhibitors have demonstrated cardiovascular and renal benefits across various patient populations; however, their role in patients undergoing TAVI remains unclear. This study is aimed at evaluating the association between SGLT2 inhibitor use and clinical outcomes in this population.

**Methods:**

We conducted a systematic review and meta‐analysis comparing SGLT2 inhibitors versus non‐SGLT2 inhibitors or standard care in patients undergoing TAVI. Major databases were searched through March 2026, with Embase updated through June 2026. Pooled analyses were performed using random‐effects models to calculate risk ratios (RRs) and mean differences (MDs) with 95% confidence intervals (CIs).

**Results:**

Five studies involving 6111 patients (SGLT2 inhibitors: *n* = 2933; control: *n* = 3178) were included. In primary random‐effects models, SGLT2 inhibitors were associated with lower all‐cause mortality (RR 0.68, 95% CI 0.48–0.97, *p* = 0.03), heart failure (HF) hospitalization (RR 0.62, 95% CI 0.42–0.90, *p* = 0.01), and major adverse cardiovascular events (RR 0.68, 95% CI 0.48–0.96, *p* = 0.03). However, in REML/Hartung–Knapp sensitivity analyses, CIs crossed the null for all‐cause mortality, HF hospitalization, MACE, and pooled hazard ratios. Mortality benefits were absent at 6 and 12 months and in the randomized trial but persisted in low left ventricular ejection fraction (RR 0.56, 95% CI 0.35–0.89, *p* = 0.01). No significant differences occurred in acute kidney injury, urinary tract infections, or echocardiographic parameters.

**Conclusion:**

SGLT2 inhibitor use was associated with lower all‐cause mortality and HF hospitalization after TAVI, but the evidence is limited by observational designs, heterogeneity, and low certainty for several outcomes. Further randomized trials are required.

## 1. Introduction

Aortic stenosis (AS) is a progressive valvular heart disease characterized by obstruction of left ventricular outflow, most commonly resulting from age‐related degenerative calcification of the aortic valve [1]. It is among the most prevalent valvular heart diseases worldwide, with its incidence steadily increasing in parallel with the aging population [2, 3]. Overall, AS accounts for nearly 43% of all valvular heart diseases, making it a leading form of valvular pathology globally [4].

The clinical course of AS is marked by a prolonged asymptomatic phase followed by rapid deterioration once symptoms develop. Symptomatic AS is associated with a poor prognosis and a markedly increased risk of mortality if left untreated [5].

Given this substantial disease burden, timely intervention is essential. In recent years, transcatheter aortic valve implantation (TAVI) has significantly transformed the management of AS. Initially developed for patients at high or prohibitive surgical risk, TAVI has emerged as an effective therapeutic option for individuals with symptomatic severe AS [6]. With continuous advances in device technology, improved patient selection, and increasing operator experience, the use of TAVI has expanded to include intermediate‐risk and even lower‐risk populations [2, 3, 7]. Contemporary valvular heart disease guidelines now recommend TAVI as a first‐line treatment option in selected patients with severe symptomatic AS [1]. Despite these improvements, heart failure (HF) and cardiovascular complications remain important determinants of prognosis following TAVI, highlighting the need for adjunctive pharmacological strategies to improve outcomes [8].

Sodium–glucose cotransporter 2 (SGLT2) inhibitors were initially developed for the management of Type 2 diabetes mellitus but have demonstrated substantial cardiovascular and renal benefits [9]. These agents significantly reduce HF–related outcomes, including hospitalizations, and lower the risk of adverse renal events in patients with T2DM [9, 10]. Accordingly, the American Diabetes Association recommends their use in patients with T2DM and HF or at high cardiovascular risk [11]. However, their use may be associated with adverse events such as genital infections, volume depletion, and rare cases of euglycemic diabetic ketoacidosis [12].

Patients undergoing TAVI remain at risk of complications, particularly HF hospitalization, mortality, and kidney‐related events. Although SGLT2 inhibitors have demonstrated benefits in HF populations, it remains unclear whether these effects extend to patients undergoing TAVI, especially when initiated before or after the procedure.

Despite growing evidence supporting SGLT2 inhibitors in cardiovascular disease, evidence specific to patients undergoing TAVI remains limited and heterogeneous. Compared with previous systematic reviews, this review includes additional eligible studies, expands the evaluated clinical and echocardiographic outcomes, provides the first quantitative synthesis of clinical outcomes in patients with low LVEF, incorporates follow‐up‐specific analyses, and carefully evaluates potentially overlapping TriNetX datasets to minimize double‐counting of participants. This study is aimed at evaluating the association between SGLT2 inhibitor use and clinical outcomes in adult patients undergoing TAVI.

## 2. Methods

### 2.1. Study Design and Registration

The study methodology and results were reported following the Preferred Reporting Items for Systematic Reviews and Meta‐Analyses (PRISMA) statement guidelines [13, 14]. The study protocol was predetermined and registered (PROSPERO: CRD420261377322).

### 2.2. Eligibility Criteria

We included randomized controlled trials (RCTs) and observational studies that met our predefined eligibility criteria: the population (P) comprised adults (≥ 18 years) with AS undergoing TAVI; the intervention (I) involved the administration of SGLT2 inhibitors before and/or after TAVI (e.g., dapagliflozin or empagliflozin), regardless of the treatment regimen; the comparison (C) was standard care without SGLT2 inhibitor administration or placebo; and the outcomes (O) included the primary outcomes of all‐cause mortality and HF hospitalization, whereas the secondary outcomes were major adverse cardiovascular events (MACEs), change in left ventricular ejection fraction (LVEF), change in left ventricular end‐diastolic diameter (LVEDD), change in left ventricular end‐systolic diameter (LVESD), acute kidney injury, and urinary tract infection. Additional safety outcomes that were not suitable for quantitative synthesis due to limited reporting were summarized narratively.

The definitions of MACE across the included studies are summarized in Table S1.

### 2.3. Search Strategy

We searched PubMed, Scopus, Web of Science, and Cochrane CENTRAL from inception to March 27, 2026. An additional search of Embase was performed from inception to June 15, 2026, to ensure comprehensive literature coverage. The search strategy combined Medical Subject Headings (MeSH) terms and free‐text keywords related to the population, intervention, and study design, and is presented in Table S2.

PubMed key search terms included: (“SGLT2 Inhibitors”[Mesh] OR “sodium glucose cotransporter 2 inhibitor ^∗^” OR SGLT2i OR SGLT2 inhibitor ^∗^ OR dapagliflozin OR empagliflozin OR canagliflozin OR ertugliflozin OR sotagliflozin OR ipragliflozin OR luseogliflozin OR remogliflozin) AND (“Transcatheter Aortic Valve Replacement” OR TAVR OR TAVI OR “Transcatheter Aortic Valve Implantation”). There were no restrictions on language or publication date. Moreover, the reference lists of included studies and pertinent systematic reviews were carefully reviewed to identify any additional relevant studies. Conference abstracts, reviews, editorials, and non‐original studies were excluded. Only peer‐reviewed published studies were considered; therefore, grey literature and preprints were not systematically searched.

### 2.4. Study Selection and Data Extraction

Search results were imported into Rayyan [15], and duplicate records were removed. Independent reviewers screened titles and abstracts in accordance with the eligibility criteria. Potentially relevant studies underwent full‐text review. Any discrepancies between the reviewers were resolved through consensus or consultation with a third reviewer.

A standardized, prepiloted data extraction form was used to systematically collect information from all included studies. Extracted data encompassed study characteristics (study ID, year of publication, study design, country, and arms), patient characteristics (sample size, age, sex, LVEF, clinical presentation such as hypotension, CAD, stroke, and AF), follow‐up duration, and MACE data. All data were extracted directly from the published manuscripts and their supporting information.

Studies with potential overlapping populations (e.g., derived from the same database, such as TriNetX) and overlapping recruitment periods were carefully evaluated to avoid duplication of data in the same analysis. Potentially overlapping studies were assessed by comparing data sources, recruitment periods, participating centers, and study populations. When overlap was suspected, the study providing the most comprehensive and clinically relevant data for the predefined outcomes of interest was retained, while the remaining studies were excluded from quantitative synthesis and considered in the discussion. Details regarding excluded studies and reasons for exclusion are provided in Table S3.

### 2.5. Quality Assessment and Certainty of Evidence

The methodological quality and risk of bias for the included studies were assessed independently by two reviewers. RCTs were assessed for bias using the revised Cochrane Risk of Bias tool (RoB 2.0) [16], assessing bias across five domains: (1) randomization process, (2) deviations from intended interventions, (3) missing outcome data, (4) measurement of outcome, and (5) selection of the reported result. Each domain was classified as low risk, some concerns, or high risk. Observational studies were assessed for bias using the Newcastle–Ottawa Quality Assessment Form for Cohort Studies (NOS) [17], assessing bias across three items: (1) selection, (2) outcome, and (3) comparability. Each item was classified as good quality, fair quality, or poor quality. In addition to the NOS assessment, we considered whether observational studies employed statistical methods to address confounding, such as propensity score matching, inverse probability of treatment weighting, or multivariable adjustment, when interpreting the certainty of evidence and overall risk of bias.

We evaluated the certainty of evidence using the GRADE approach based on risk of bias, inconsistency, indirectness, imprecision, and publication bias [18, 19]. Given that only a single RCT was available, GRADE assessments were restricted to observational studies, as certainty ratings based on a single randomized study would provide limited informative value for evidence grading.

### 2.6. Data Synthesis and Statistical Analysis

We used Review Manager (RevMan) software to pool the data of the included trials [20] and R (Version 4.4.2, R Core Team, 2024) [21]. We calculated the risk ratio (RR) for dichotomous outcomes using the Mantel–Haenszel method and mean difference (MD) for continuous outcomes using the inverse variance method, along with 95% confidence intervals (CIs). For echocardiographic outcomes (LVEF, LVEDD, and LVESD), pooled analyses were performed using change‐from‐baseline values. The mean changes and their corresponding standard deviations were directly reported by Paolisso et al. [22] and Keskin et al. [23]. For Fan et al. [24], change‐from‐baseline standard deviations were not reported; therefore, they were calculated according to Cochrane Handbook guidance using baseline and follow‐up standard deviations with an assumed correlation coefficient of 0.5. Posttreatment values were not pooled with change‐from‐baseline values within the same analysis. Additionally, we extracted and pooled the hazard ratios (HRs) for primary outcomes. Adjusted HRs were extracted from observational studies, whereas crude HRs were used as surrogates for RCTs. We pooled HR using the generic inverse variance method. To handle differences in study settings and participant demographics, we used a random‐effects model to address potential heterogeneity. Between‐study variance (tau‐squared) was calculated using the DerSimonian and Laird method [25]. We assessed heterogeneity using the *I* ^2^ statistic, which gauges the part of total variability due to heterogeneity instead of random chance. As per the Cochrane Handbook, the chi‐square test was evaluated as significant heterogeneity if the alpha level was less than 0.1, whereas the *I* ^2^ test was interpreted as follows: not significant was indicated by 0%–40%, moderate heterogeneity was indicated by 30%–60%, and substantial heterogeneity was indicated by 50%–90%. If the *p* value was less than 0.05, the total effect size was regarded as statistically significant. Publication bias was not assessed because of the inclusion of fewer than 10 studies, as recommended by Egger et al. [26].

Additional analyses were conducted in patients with low LVEF, as defined by each study. Outcomes were analyzed at the longest available follow‐up for each study. Subgroup analyses were also performed according to study design and follow‐up duration (6 months and 1 year), depending on data availability; these findings are consistent. A systematic leave‐one‐out sensitivity analysis was performed by iteratively omitting one study at a time to assess the stability of the pooled estimates and identify studies driving between‐study heterogeneity. We also conducted a sensitivity analysis by using the restricted maximum‐likelihood (REML) estimator for between‐study variance (tau‐squared) and Hartung–Knapp (HK) adjustment for the random‐effect model.

## 3. Results

### 3.1. Study Selection

A comprehensive search of five major databases identified 459 records. After the removal of 186 duplicates, 273 records remained for title and abstract screening. Of these, 261 records were excluded for not meeting the eligibility criteria. Twelve full‐text articles were assessed for eligibility, and seven were excluded following full‐text review because of inappropriate intervention (*n* = 3), conference abstract‐only publications (*n* = 1), and potential overlapping populations (*n* = 3). Details regarding excluded overlapping studies and reasons for exclusion are provided in Table S3. Ultimately, five studies met all predefined inclusion criteria and were included in the final analysis. The study selection process is presented in the PRISMA flow diagram (Figure 1).

**Figure 1:**
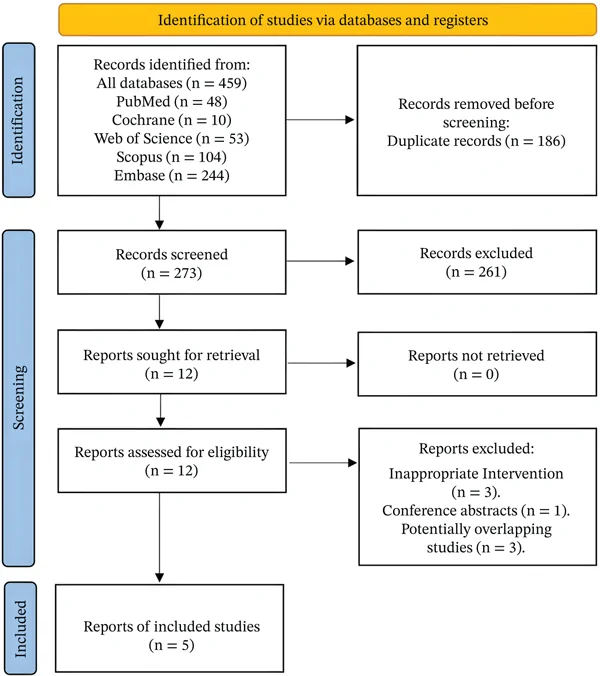
PRISMA flow diagram of the study selection process.

### 3.2. Study Characteristics

Five studies were included [22–24, 27, 28], comprising a total of 6111 patients undergoing TAVI with or without SGLT2 inhibitor therapy. Among them, 2933 patients received SGLT2 inhibitors, whereas 3178 patients were assigned to the control or standard care groups. A summary and baseline characteristics of the included studies are in Table 1. The detailed inclusion and exclusion criteria are presented in Table S4.

**Table 1:** Summary and baseline characteristics of included studies.

Study ID	Country	Study Design	Population	Follow up (months)	Arms	Patients, *n*	Age, mean ± SD	Male, *n* (%)	LVEF, mean ± SD	Clinical presentation			
										Hypertension, *n* (%)	CAD, *n* (%)	Stroke, *n* (%)	AF, *n* (%)
Keskin et al. 2026 [25]	Türkiye	Retrospective cohort study	Patients with LVEF < 50% undergoing transfemoral TAVI for severe aortic stenosis	37.3	SGLT2i Users	78	75.19 ± 9.51	49 (62.8)	37.22 ± 8.99	65 (83.3%)	50 (64.1%)	1 (1.3%)	33 (42.3%)
					Non‐SGLT2i Users	148	76.76 ± 9.15	84 (56.8)	41.96 ± 9.14	128 (86.5%)	89 (60.1%)	11 (7.4%)	54 (36.5%)
Yasmin et al. 2026 [24]	Multicenter	Retrospective cohort study	CHF patients undergoing TAVR for aortic stenosis	12	SGLT2i (HFpEF)	1380	76.0 ± 9.0	744 (53.9%)		1310 (94.9%)	1204 (87.2%)	500 (36.2%)	384 (27.8%)
					SGLT2i (HFrEF)	754	76.0 ± 9.0	438 (58.1%)	41.7 ± 15.7	706 (93.6%)	674 (89.4%)	368 (48.8%)	258 (34.2%)
					Non‐SGLT2i (HFpEF)	1380	76.0 ± 9.0	744 (53.9%)		1310 (94.9%)	1204 (87.2%)	500 (36.2%)	384 (27.8%)
					Non‐SGLT2i (HFrEF)	754	76.0 ± 9.0	438 (58.1%)	45.2 ± 17.5	706 (93.6%)	674 (89.4%)	368 (48.8%)	258 (34.2%)
Raposeiras‐Roubin et al. 2025 [26]	Spain	Randomized controlled trial	Patients with severe aortic stenosis undergoing TAVI.	12	Dapagliflozin	605	82.4 ± 5.6	306 (50.6%)	54.9 ± 12.3	518 (85.6%)	237 (39.2%)	61 (10.1%)	250 (41.3%)
					Standard care	617	82.4 ± 5.5	312 (50.6%)	54.8 ± 12.1	519 (84.0%)	237 (39.2%)	69 (11.2%)	274 (44.3%)
Paolisso et al. 2024 [27]	Multicenter	Retrospective cohort study	T2DM patients with severe AS, LVEF < 50%, and EVCD undergoing TAVI	24	SGLT2i	74	77 ± 6.04	58 (78.4%)	34.33 ± 12.09	58 (78.4%)	53 (71.6%)	—	41 (55.4%)
					No‐SGLT2i	237	80.66 ± 5.22	150 (63.3%)	38.66 ± 9.69	203 (85.7%)	146 (61.6%)	—	95 (40.1%)
Fan et al. 2024 [23]	China	Prospective cohort study	Non‐diabetic severe AS undergoing TAVR	6	Dapagliflozin	42	74.23 ± 5.14	22 (52.4%)	50.57 ± 9.24	28 (66.7%)	24 (57.1%)	—	—
					Conventional therapy	42	72.64 ± 5.27	21 (50.0%)	49.00 ± 11.21	27 (64.3%)	25 (59.5%)	—	—

Abbreviations: AF, atrial fibrillation; AS, aortic stenosis; CAD, coronary artery disease; CHF, congestive heart failure; EVCD, extravalvular cardiac damage; HFpEF, heart failure with preserved ejection fraction; HFrEF, heart failure with reduced ejection fraction; LVEF, left ventricular ejection fraction; n, number of patients; SD, standard deviation; SGLT2i, sodium–glucose cotransporter 2 inhibitors; T2DM, Type 2 diabetes mellitus; TAVI, transcatheter aortic valve implantation; TAVR, transcatheter aortic valve replacement.

### 3.3. Quality Assessment and Certainty of Evidence

Methodological quality was assessed using tools appropriate to the study design. The RCT by Raposeiras‐Roubin et al. [28] was judged to have an overall rating of some concerns due to potential bias arising from deviations from intended interventions, as the study employed a single‐blinded design (Figure S1).

The four observational studies (Paolisso et al., Yasmin et al., Fan et al., and Keskin et al.) [22–24, 27] were assessed using the NOS, which evaluates selection, comparability, and outcome domains. Paolisso et al., Yasmin et al., and Keskin et al. each received a score of 9/9, whereas Fan et al. received a score of 7/9, indicating generally high methodological quality among the included observational studies (Table S5). Several studies employed statistical methods to address confounding, including propensity score matching, inverse probability of treatment weighting, or multivariable adjustment. Nevertheless, residual confounding inherent to observational study designs cannot be completely excluded. The certainty of evidence for each outcome in the overall TAVI population was assessed and is summarized in Table 2. The certainty of evidence according to follow‐up duration is summarized in Table S6.

**Table 2:** GRADE certainty of evidence from observational studies for overall TAVI and low EF outcomes.

Population	Outcomes	Anticipated absolute effects (95% CI)		Relative effect (95% CI)	№ of participants (studies)	Certainty of the evidence (GRADE)
		Risk with [non SGLT2]	Risk with [SGLT2]			
Overall	All‐cause mortality	102 per 1000	61 per 1000 (37 to 101)	RR 0.60 (0.36 to 0.99)	4586 (3 nonrandomized studies)	⨁◯◯◯ Very low^b^
	HF hospitalization	177 per 1000	88 per 1000 (42 to 185)	RR 0.50 (0.24 to 1.05)	537 (2 nonrandomized studies)	⨁◯◯◯ Very low^a^
	MACE	331 per 1000	205 per 1000 (119 to 357)	RR 0.62 (0.36 to 1.08)	4615 (3 non‐randomized studies)	⨁◯◯◯ Very low^b,c^
	AKI	210 per 1000	103 per 1000 (27 to 392)	RR 0.49 (0.13 to 1.87)	4304 (2 nonrandomized studies)	⨁◯◯◯ Very low^a,b^
	LVEF Change	The mean change in LVEF was 5.71%	MD 1.5% higher (1.49 lower to 4.49 higher)		621 (3 nonrandomized studies)	⨁◯◯◯ Very low^a,b^
	LVESD Change	The mean change in LVESD was −1.37 mm	MD 2.5 mm lower (7.81 lower to 2.81 higher)		310 (2 nonrandomized studies)	⨁◯◯◯ Very low^a,b^
	LVEDD Change	The mean change in LVEDD was −1.13 mm	MD 2.66 mm lower (8.02 lower to 2.7 higher)		310 (2 nonrandomized studies)	⨁◯◯◯ Very low^a,b^

Low LVEF	All‐cause Mortality	255 per 1000	143 per 1000 (89 to 227)	RR 0.56 (0.35 to 0.89)	2, *n* (3 observational studies)	⨁◯◯◯ Very low^b^
	HF Hospitalization	177 per 1000	89 per 1000 (42 to 186)	RR 0.50 (0.24 to 1.05)	537 (2 observational studies)	⨁◯◯◯ Very low^a^
	MACE	407 per 1000	248 per 1000 (147 to 423)	RR 0.61 (0.36 to 1.04)	2045 (3 observational studies)	⨁◯◯◯ Very low^b,c^
	AKI	234 per 1000	119 per 1000 (28 to 505)	RR 0.51 (0.12 to 2.16)	1734 (2 observational studies)	⨁◯◯◯ Very low^a,b^

*Note:* The risk in the intervention group (and its 95% confidence interval) is based on the assumed risk in the comparison group and the relative effect of the intervention (and its 95% CI).

Abbreviations: AKI, acute kidney injury; CI, confidence interval; EF, ejection fraction; HF, heart failure; LVEDD, left ventricular end‐diastolic diameter; LVEF, left ventricular ejection fraction; LVESD, left ventricular end‐systolic diameter; MACE, major adverse cardiovascular events; MD, mean difference; RR, risk ratio; TAVI, transcatheter aortic valve implantation.

^a^Downgraded for imprecision due to the limited number of studies.

^b^Downgraded for inconsistency due to very high heterogeneity.

^c^Downgraded for indirectness due to it being a composite outcome that had different definitions 736 across the included studies.

### 3.4. Primary Outcomes

#### 3.4.1. All‐Cause Mortality

Primary pooled analysis (all studies combined): In a preliminary analysis of four studies (*n* = 5808), SGLT2 inhibitors were associated with a significant reduction in all‐cause mortality (RR = 0.68, 95% CI: 0.48 to 0.97; *p* = 0.03), albeit with substantial heterogeneity (*I* ^2^ = 74%) (Figure 2a).

**Figure 2:**
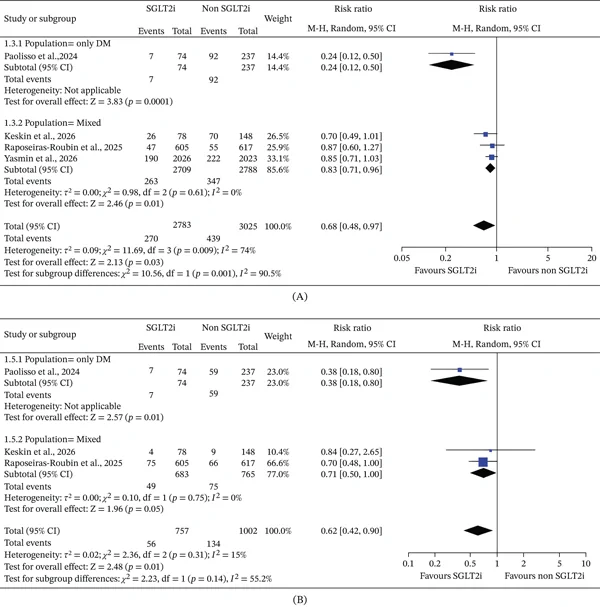
Forest plots of the overall TAVI population. (A) All‐cause mortality. (B) HF hospitalization.

##### 3.4.1.1. Findings by Study Design

When stratified, the reduction was not significant in the RCT (RR = 0.87, 95% CI: 0.60 to 1.27; *p* = 0.47), but was significant in observational studies (RR = 0.60, 95% CI: 0.36 to 0.99; *p* = 0.05; *I* ^2^ = 82%) (Figure S2).

Pooled adjusted HRs were associated with lower mortality (HR = 0.78, 95% CI: 0.63 to 0.98; *p* = 0.03; *I* ^2^ = 27%) (Figure S3).

##### 3.4.1.2. Follow‐Up Duration

No significant difference was observed at 6 months (3 studies, *n* = 5497: RR = 0.83, 95% CI: 0.67 to 1.02; *p* = 0.08; *I* ^2^ = 0%) (Figure S4) or at 12 months (3 studies, *n* = 5582: RR = 0.74, 95% CI: 0.48 to 1.15; *p* = 0.18; *I* ^2^ = 68%) (Figure S5).

##### 3.4.1.3. Low LVEF Subgroup

In patients with low LVEF (3 studies, *n* = 2034), SGLT2 inhibitors significantly reduced mortality (RR = 0.56, 95% CI: 0.35 to 0.89; *p* = 0.01; *I* ^2^ = 75%) (Figure 3a).

**Figure 3:**
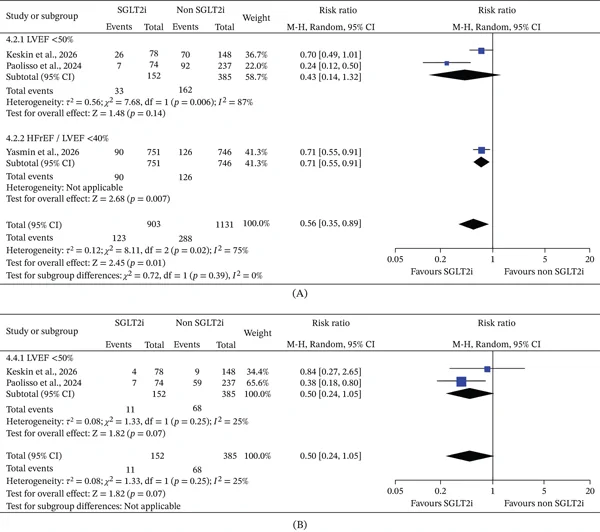
Forest plots of the low EF TAVI population. (A) All‐cause mortality. (B) HF hospitalization.

#### 3.4.2. HF Hospitalization

Primary pooled analysis: In a preliminary analysis of 3 studies (*n* = 1759), SGLT2 inhibitors significantly reduced HF hospitalization (RR = 0.62, 95% CI: 0.42 to 0.90; *p* = 0.01; *I* ^2^ = 15%) (Figure 2b).

##### 3.4.2.1. Findings by Study Design

The effect was borderline in the RCT (RR = 0.70, 95% CI: 0.48 to 1.00; *p* = 0.05) and nonsignificant in observational studies (RR = 0.50, 95% CI: 0.24 to 1.05; *p* = 0.07; *I* ^2^ = 25%) (Figure S6).

Pooled adjusted HRs were associated with lower HF hospitalization (HR = 0.58, 95% CI: 0.35 to 0.94; *p* = 0.03; *I* ^2^ = 58%) (Figure S7).

##### 3.4.2.2. Follow‐Up Duration

The benefit remained significant at 12 months (2 studies, *n* = 1533: RR = 0.65, 95% CI: 0.47 to 0.91; *p* = 0.01; *I* ^2^ = 0%) (Figure S8).

##### 3.4.2.3. Low LVEF Subgroup

No significant difference was observed in patients with low LVEF (2 studies, *n* = 537: RR = 0.50, 95% CI: 0.24 to 1.05; *p* = 0.07; *I* ^2^ = 25%) (Figure 3b).

### 3.5. Secondary Outcomes

#### 3.5.1. MACE

Primary pooled analysis: In a preliminary analysis of 4 studies (*n* = 5837), SGLT2 inhibitors were associated with a significant reduction in MACE (RR = 0.68, 95% CI: 0.48 to 0.96; *p* = 0.03), but with substantial heterogeneity (*I* ^2^ = 86%) (Figure 4a).

**Figure 4:**
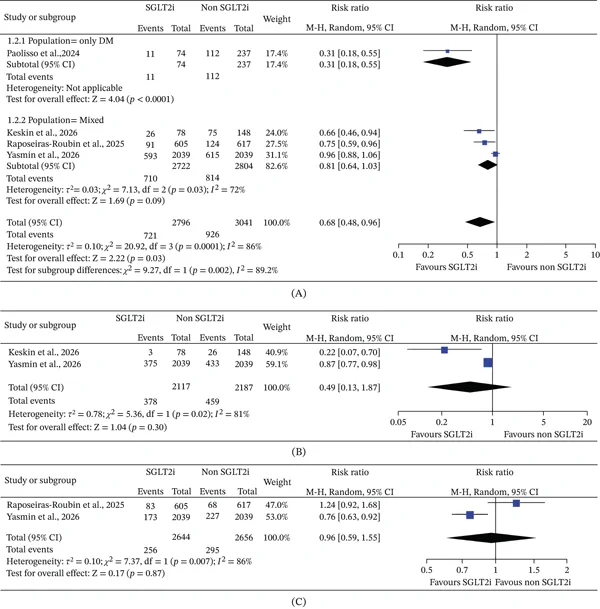
Forest plots of the overall TAVI population. (A) MACE. (B) AKI. (C) Urinary tract infection.

##### 3.5.1.1. Findings by Study Design

The reduction was significant in the RCT (RR = 0.75, 95% CI: 0.59 to 0.96; *p* = 0.02), but not in observational studies (RR = 0.62, 95% CI: 0.36 to 1.08; *p* = 0.09; *I* ^2^ = 89%) (Figure S9).

##### 3.5.1.2. Follow‐Up Duration

No significant differences were observed at 6 months (2 studies, *n* = 4389: RR = 0.52, 95% CI: 0.13 to 2.02; *p* = 0.34; *I* ^2^ = 83%) (Figure S10) or at 12 months (3 studies, *n* = 5611: RR = 0.90, 95% CI: 0.76 to 1.07; *p* = 0.22; *I* ^2^ = 44%) (Figure S11).

##### 3.5.1.3. Low LVEF Subgroup

No significant difference was found (4 studies, *n* = 2668: RR = 0.69, 95% CI: 0.47 to 1.02; *p* = 0.06; *I* ^2^ = 81%) (Figure S12).

#### 3.5.2. AKI

No significant difference was observed between SGLT2 inhibitor and non‐SGLT2 inhibitor groups in the overall analysis (2 studies, *n* = 4304) (RR = 0.49, 95% CI: 0.13 to 1.87; *p* = 0.30; *I* ^2^ = 81%) or in low LVEF patients (2 studies, *n* = 1734) (RR = 0.51, 95% CI: 0.12 to 2.16; *p* = 0.36; *I* ^2^ = 84%) (Figures 4b and S13).

#### 3.5.3. Urinary Tract Infection

At 12 months (2 studies, *n* = 5300), no significant difference was observed between groups (RR = 0.96, 95% CI: 0.59 to 1.55; *p* = 0.87; *I* ^2^ = 86%) (Figure 4c).

#### 3.5.4. Change in LVEF

No significant difference was observed between SGLT2 inhibitor and non‐SGLT2 inhibitor groups in the overall analysis (3 studies, *n* = 621) (MD = 1.50, 95% CI: −1.49 to 4.49; *p* = 0.33; *I* ^2^ = 80%) or at 6 months (2 studies, *n* = 310) (MD = −0.20, 95% CI: −1.83 to 1.44; *p* = 0.81; *I* ^2^ = 0%) (Figures 5a and S14).

**Figure 5:**
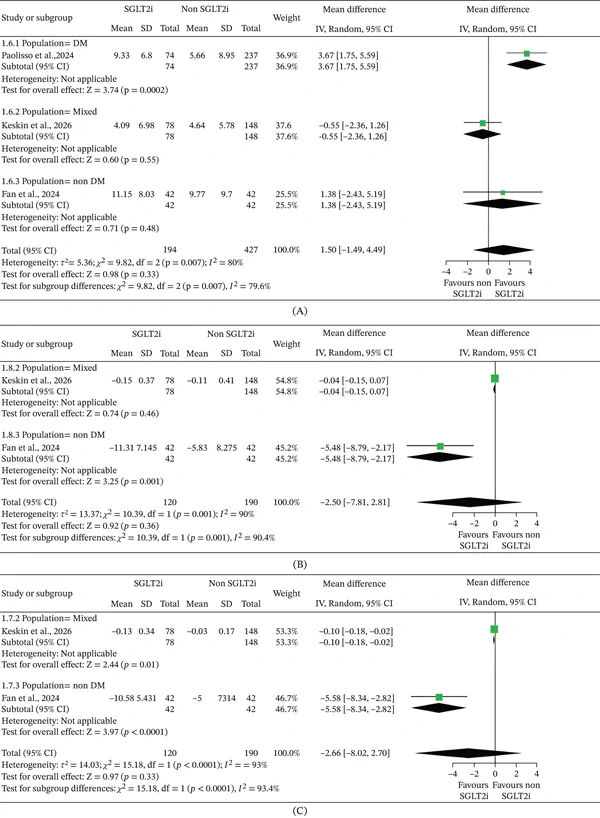
Forest plots of the overall TAVI population. (A) LVEF. (B) LVESD. (C) LVEDD.

In patients with LVEF < 50% (2 studies, *n* = 537), no significant difference was observed between the SGLT2 inhibitor and non‐SGLT2 inhibitor groups (MD = 1.55, 95% CI: −2.59 to 5.68; *p* = 0.46; *I* ^2^ = 90%) (Figure S15).

#### 3.5.5. Change in LVESD

No significant difference was observed between the SGLT2 inhibitor and non‐SGLT2 inhibitor groups (2 studies, *n* = 310) (MD = −2.50, 95% CI: −7.81 to 2.81; *p* = 0.36; *I* ^2^ = 90%) (Figure 5b).

#### 3.5.6. Change in LVEDD

No significant difference was observed between the SGLT2 inhibitor and non‐SGLT2 inhibitor groups (2 studies, *n* = 310) (MD = −2.66, 95% CI: −8.02 to 2.70; *p* = 0.33; *I* ^2^ = 93%) (Figure 5c).

### 3.6. Additional Safety Outcomes (Narrative Summary)

Additional safety outcomes, including genital infection, hypotension, syncope, major hypoglycemia, nontraumatic amputation, and cancer, were reported only in the study by Raposeiras‐Roubin et al. 2025 (DapaTAVI trial) [28] and therefore could not be quantitatively synthesized. These outcomes are summarized in Table S7.

### 3.7. Sensitivity Analysis

Sensitivity analyses were performed using an iterative leave‐one‐out approach to assess the stability of the pooled estimates and identify influential studies. This identified Paolisso et al. 2004 as a major source of between‐study variability. For all‐cause mortality, excluding Paolisso et al. 2024 maintained statistical significance with no heterogeneity (RR = 0.83, 95% CI: 0.71 to 0.96; *I* ^2^ = 0%), whereas the 12‐month analysis remained nonsignificant (RR = 0.86, 95% CI: 0.73 to 1.01; *I* ^2^ = 0%) (Figures S16 and S17). In the low LVEF subgroup, the effect remained significant with reduced heterogeneity (RR = 0.71, 95% CI: 0.58 to 0.87; *I* ^2^ = 0%) (Figure S18).

For HF hospitalization, exclusion of Paolisso et al. 2024 yielded consistent results with borderline significance and no heterogeneity (RR = 0.71, 95% CI: 0.50 to 1.00; *I* ^2^ = 0%) (Figure S19).

For MACE, excluding Paolisso et al. 2024 attenuated the overall effect to nonsignificance (RR = 0.81, 95% CI: 0.64 to 1.03; *I* ^2^ = 72%), and in the low LVEF subgroup (RR = 0.86, 95% CI: 0.70 to 1.05; *I* ^2^ = 37%) (Figures S20 and S21). Additionally, exclusion of Raposeiras‐Roubin et al. 2025 in the 12‐month analysis resulted in consistent nonsignificant findings with no heterogeneity (RR = 0.96, 95% CI: 0.88 to 1.06; *I* ^2^ = 0%) (Figure S22).

For LVEF, exclusion of Paolisso et al., 2024 confirmed nonsignificant results with elimination of heterogeneity (MD = −0.20, 95% CI: −1.83 to 1.44; *I* ^2^ = 0%) (Figure S23).

Notably, sensitivity analyses demonstrated that exclusion of the DM‐only study by Paolisso et al. consistently reduced heterogeneity across most outcomes, suggesting that differences in patient populations may partially explain the observed variability in the pooled estimates.

Additional sensitivity analyses using the REML estimator with Hartung–Knapp adjustment yielded more conservative estimates. Under this approach, the CIs for all‐cause mortality, HF hospitalization, MACE, and the corresponding HR analyses crossed the null value, attenuating the statistical significance observed in the primary analyses. These findings suggest that the observed associations are sensitive to the choice of meta‐analytic model and should therefore be interpreted with caution. Detailed results of the REML/Hartung–Knapp analyses are provided in Tables S8–S10.

## 4. Discussion

In this systematic review and meta‐analysis, we evaluated the association between SGLT2 inhibitor use and clinical outcomes in patients undergoing TAVI, a population that remains at considerable risk for adverse cardiovascular and renal outcomes despite procedural advancements. Our findings showed an association between SGLT2 inhibitor use and lower all‐cause mortality and HF hospitalization compared with non‐SGLT2 inhibitor or standard care groups. However, the association with lower all‐cause mortality was not consistently observed across follow‐up‐specific analyses and should therefore be interpreted with caution. The mortality signal appeared stronger in patients with low LVEF, although other outcomes in this subgroup remained uncertain, suggesting a potential role for SGLT2 inhibitors in higher‐risk subgroups.

In contrast, findings regarding MACE were inconsistent across analyses. Although the overall analysis demonstrated a statistically significant association with lower MACE rates in the SGLT2 inhibitor group, this finding was not consistently observed across follow‐up‐specific, subgroup, and sensitivity analyses and was accompanied by substantial heterogeneity. Furthermore, the definition of MACE varied across the included studies, ranging from mortality and HF–related events to broader composite cardiovascular outcomes that additionally included myocardial infarction and stroke. Therefore, the pooled estimate should be interpreted as a study‐defined composite cardiovascular outcome rather than a uniformly defined clinical endpoint.

Similarly, findings for AKI, urinary tract infection, and echocardiographic parameters remained largely nonsignificant across subgroup and sensitivity analyses. Some effects varied across subgroup analyses, particularly by follow‐up duration and study design, suggesting potential heterogeneity in treatment response. Notably, exclusion of the DM‐only study (Paolisso et al.) reduced heterogeneity across most outcomes, which may partially explain the observed variability, as underlying metabolic differences may influence treatment effects.

Valvular heart disease guidelines, including the 2025 ESC/EACTS guidelines, focus on procedural strategies, and optimal medical therapy in patients undergoing TAVI but do not provide specific recommendations regarding SGLT2 inhibitors in this population [1]. In contrast, both the 2021 ESC HF guidelines and the 2022 AHA/ACC/HFSA guidelines recommend SGLT2 inhibitors as cornerstone therapy for HF, regardless of diabetes status, because of consistent reductions in HF hospitalization and cardiovascular mortality [29, 30]. In this context, our findings suggest that patients undergoing TAVI who also have HF may represent a clinically relevant population for further evaluation of SGLT2 inhibitors, particularly regarding reductions in mortality and HF hospitalization.

Observational studies using large real‐world datasets have reported similar findings. Morel et al. and Obeidat et al. demonstrated significant reductions in all‐cause mortality among TAVI patients receiving SGLT2 inhibitors, with HRs of 0.83 (95% CI: 0.71–0.97) and up to 0.59 at long‐term follow‐up, respectively [31, 32]. However, these studies were excluded from our pooled analysis because of overlapping populations from the same research network (TriNetX).

Importantly, the DapaTAVI trial, the only randomized trial included in this review, demonstrated that dapagliflozin significantly reduced the composite endpoint of all‐cause death or worsening HF (HR 0.72, 95% CI: 0.55–0.95), with the observed benefit being primarily driven by reductions in worsening HF events rather than a clear independent reduction in all‐cause mortality [28]. Given that DapaTAVI represents the only randomized evidence currently available, its findings should be considered particularly informative when interpreting potential causal effects of SGLT2 inhibitors in the post‐TAVI setting. Consistent with these findings, our subgroup analysis showed that mortality benefits were less apparent in randomized data, whereas HF hospitalization demonstrated a more robust signal. Similarly, a meta‐analysis by Daniyal et al. demonstrated significant reductions in both mortality (HR 0.72, 95% CI: 0.53–0.96) and HF hospitalization (HR 0.74, 95% CI: 0.61–0.90) following TAVI [33].

Importantly, sensitivity analyses using the REML estimator with Hartung–Knapp adjustment resulted in CIs crossing the null for all‐cause mortality, HF hospitalization, MACE, and the corresponding HR analyses, suggesting that these findings are not robust to more conservative meta‐analytic assumptions. Therefore, the observed associations should be interpreted cautiously and considered hypothesis‐generating rather than definitive evidence of treatment benefit.

Beyond the TAVI setting, large HF meta‐analyses consistently demonstrate class‐wide benefits of SGLT2 inhibitors. Vaduganathan et al. reported reductions in cardiovascular death and HF hospitalization across a wide range of ejection fractions [34], whereas Fukuta et al., focusing on HFpEF, showed a significant reduction in the composite of HF hospitalization and cardiovascular death (HR 0.78, 95% CI: 0.70–0.87) [35]. Patel et al. further demonstrated a modest reduction in MACEs (HR 0.91, 95% CI: 0.87–0.96), largely driven by cardiovascular death [36]. Collectively, these findings are consistent with our results, where benefits were more consistently observed for HF–related endpoints rather than broader, heterogeneous composite cardiovascular outcomes such as MACE.

Emerging evidence also suggests that these benefits extend beyond transcatheter interventions. In patients undergoing surgical aortic valve replacement, Jacquemyn et al. demonstrated lower long‐term mortality (13.8% vs. 25.8%, *p* = 0.012) and reduced stroke risk, suggesting a potential class effect across valve replacement strategies [37].

From a mechanistic perspective, these findings align with the concept of a comprehensive cardiometabolic approach. As described by Karakasis et al., AS involves complex interactions between hemodynamic stress, inflammation, fibrosis, and metabolic dysregulation [38]. SGLT2 inhibitors may modulate these pathways, including myocardial energetics and inflammatory signaling, providing a plausible explanation for the observed improvements in HF outcomes, particularly in patients with reduced LVEF.

These findings support further evaluation of the potential role for SGLT2 inhibitors as adjunctive therapy in patients undergoing TAVI, particularly those with HF or reduced ejection fraction. Their association with lower mortality and HF hospitalization suggests potential benefit in this high‐risk population. However, given the predominance of observational evidence and the lack of a significant mortality benefit in the RCT subgroup, these findings should be interpreted with caution and considered hypothesis‐generating. Although the available evidence suggests potential benefit, routine use specifically for post‐TAVI risk reduction requires confirmation in adequately powered randomized trials.

This study represents one of the most comprehensive analyses evaluating SGLT2 inhibitors in patients undergoing TAVI, incorporating both randomized and real‐world evidence. We performed detailed subgroup analyses by study design, follow‐up duration, and LVEF, allowing for a more refined assessment across clinically relevant populations. Additionally, the inclusion of key clinical outcomes and the use of rigorous methodology (PRISMA, risk of bias, and GRADE) enhance the validity and clinical applicability of our findings.

### 4.1. Limitations

First, the limited number of included studies, with a predominance of observational designs, introduces the potential for residual confounding and selection bias. Although several observational studies employed propensity score matching, inverse probability of treatment weighting, or multivariable adjustment, residual confounding inherent to nonrandomized studies cannot be completely excluded. Second, heterogeneity across studies in patient characteristics, follow‐up durations, timing of SGLT2 inhibitor initiation (pre‐TAVI vs. post‐TAVI), and variability in MACE definitions may have influenced the pooled estimates and limited the interpretability of treatment effects regarding this outcome. In particular, follow‐up durations varied substantially across studies, ranging from approximately 12 to more than 37 months. As the primary RR analyses were based on cumulative event counts, pooling studies with different follow‐up periods may have influenced the estimated treatment effects despite the use of random‐effects models.

In addition, the use of large research network databases may have introduced overlapping populations, although efforts were made to minimize duplication. Furthermore, several outcomes were derived from a limited number of studies, reducing statistical power and increasing uncertainty. The certainty of evidence, as assessed by GRADE, ranged from very low to low for several key outcomes. To address differences in follow‐up duration, we performed separate 12‐month and time‐to‐event HR analyses; however, these findings should still be interpreted with caution.

Finally, the lack of individual patient‐level data precluded more detailed subgroup analyses, and publication bias could not be formally assessed because of the limited number of included studies.

### 4.2. Future Research Directions

Future studies are needed to further define the role of SGLT2 inhibitors in patients undergoing TAVI. Well‐designed, large‐scale RCTs are required to confirm the observed benefits and to clarify the optimal timing of initiation (preprocedural vs. postprocedural). In addition, studies focusing on high‐risk subgroups, particularly patients with reduced LVEF, are warranted to better identify populations most likely to benefit. Further research should also explore long‐term outcomes, including valve durability and recurrent HF, as well as potential differences between individual SGLT2 inhibitors. Finally, mechanistic studies investigating the cardiometabolic and myocardial remodeling effects of SGLT2 inhibitors in the post‐TAVI setting may provide deeper insights into their therapeutic potential.

## 5. Conclusion

In this systematic review and meta‐analysis, SGLT2 inhibitor use was associated with lower all‐cause mortality and HF hospitalization in patients undergoing TAVI, with a possible mortality signal in patients with reduced ejection fraction. Nevertheless, the observed mortality association should be interpreted cautiously, as neither the 6‐month nor the 12‐month analyses nor the only randomized trial demonstrated an independent mortality benefit. No statistically significant differences were observed between groups for other outcomes, including MACE, renal complications, and echocardiographic parameters. Sensitivity analyses using the REML estimator with Hartung–Knapp adjustment yielded more conservative estimates, with CIs crossing the null for all‐cause mortality, HF hospitalization, and MACE. Further adequately powered RCTs are warranted to confirm the efficacy and safety of SGLT2 inhibitors in this setting.

**Nomenclature**
AF: atrial fibrillation
AHA/ACC/HFSA: American Heart Association/American College of Cardiology/Heart Failure Society of America
AKI: acute kidney injury
AS: aortic stenosis
CAD: coronary artery disease
CI: confidence interval
DM: diabetes mellitus
EACTS: European Association for Cardio‐Thoracic Surgery
ESC: European Society of Cardiology
GRADE: Grading of Recommendations Assessment, Development and Evaluation
HF: heart failure
HFpEF: heart failure with preserved ejection fraction
HFrEF: heart failure with reduced ejection fraction
LVEDD: left ventricular end‐diastolic diameter
LVEF: left ventricular ejection fraction
LVESD: left ventricular end‐systolic diameter
MACE: major adverse cardiovascular events
MD: mean difference
MeSH: Medical Subject Headings
NOS: Newcastle–Ottawa Scale
PRISMA: Preferred Reporting Items for Systematic Reviews and Meta‐Analyses
PROSPERO: International Prospective Register of Systematic Reviews
RCT: randomized controlled trial
RevMan: Review Manager
RR: risk ratio
SAVR: surgical aortic valve replacement
SGLT2: sodium–glucose cotransporter 2
SGLT2i: sodium–glucose cotransporter 2 inhibitors
T2DM: Type 2 diabetes mellitus
TAVI: transcatheter aortic valve implantation

## Author Contributions

A.A.M. contributed to the study idea, conceptualization, literature screening, data extraction, statistical analysis, and manuscript writing. B.H.H. contributed to literature screening, data extraction, GRADE assessment, and manuscript writing. T.K.D. contributed to quality assessment, literature screening, data extraction, and manuscript writing. M.E.A. contributed to data analysis and manuscript writing. G.A.S. and A.S.G. contributed to literature screening, data extraction, and manuscript writing. M.A.M. contributed to data extraction, data curation, and manuscript writing. A.M.H., A.S.S., and Y.R. contributed to statistical analysis and data synthesis. O.M.A. and T.Z.S. contributed to the risk of bias assessment and GRADE certainty of evidence evaluation. M.D. and A.S.S. contributed to validation, manuscript writing, and project administration. All authors contributed to data interpretation.

## Funding

No funding was received for this manuscript.

## Disclosure

All authors critically revised the manuscript and approved the final version.

## Ethics Statement

The authors have nothing to report.

## Ethics Statement

This study is a systematic review and meta‐analysis of previously published data and does not include any individual patient data.

## Conflicts of Interest

The authors declare no conflicts of interest.

## Supporting Information

Additional supporting information can be found online in the Supporting Information section.

## Supporting information


**Supporting Information 1** Figure S1: Traffic light plot of risk of bias assessment using the Cochrane Risk of Bias 2 tool for randomized controlled trials. Figure S2: Forest plot of subgroup analysis by study design for all‐cause mortality in the overall population. Figure S3: Forest plot of pooled adjusted hazard ratios for all‐cause mortality. Figure S4: Forest plot of all‐cause mortality of the overall population at 6 months follow‐up. Figure S5: Forest plot of all‐cause mortality of the overall population at 12 months follow‐up. Figure S6: Forest plot of subgroup analysis by study design for HF hospitalization in the overall population. Figure S7: Forest plot of pooled adjusted hazard ratios for HF hospitalization. Figure S8: Forest plot of HF hospitalization of the overall population at 12 months follow‐up. Figure S9: Forest plot of subgroup analysis by study design for MACE in the overall population. Figure S10: Forest plot of MACE of the overall population at 6 months follow‐up. Figure S11: Forest plot of MACE of the overall population at 12 months follow‐up. Figure S12: Forest plot of MACE with low EF population. Figure S13: Forest plot of AKI with low EF population. Figure S14: Forest plot of LVEF of the overall population at 6 months follow‐up. Figure S15: Forest plot of LVEF with LVEF < 50% population. Figure S16: Sensitivity analysis forest plot of all‐cause mortality of the overall population. Figure S17: Sensitivity analysis forest plot of all‐cause mortality of the overall population at 12 months. Figure S18: Sensitivity analysis forest plot of all‐cause mortality with low EF population. Figure S19: Sensitivity analysis forest plot of HF hospitalization of the overall population. Figure S20: Sensitivity analysis forest plot of MACE of the overall population. Figure S21: Sensitivity analysis forest plot of MACE with low EF population. Figure S22: Sensitivity analysis forest plot of MACE of the overall population at 12 months. Figure S23: Sensitivity analysis forest plot of LVEF of the overall population. Table S1: MACE Definition (composite endpoint) across included studies. Table S2: Summary of the search strategy for all the databases. Table S3: Full‐text studies excluded due to potential population overlap and reasons for exclusion. Table S4: Inclusion and exclusion criteria of included studies. Table S5: Newcastle–Ottawa Scale assessment of observational studies. Table S6: GRADE certainty of evidence from observational studies in the overall TAVI population by follow‐up duration (12 months and 6 months). Table S7: Additional safety outcomes reported in the DapaTAVI trial not included in quantitative synthesis. Table S8: Sensitivity analysis using the REML estimator with Hartung–Knapp adjustment for overall analysis of all outcomes. Table S9: Sensitivity analyses of subgroup and follow‐up outcomes using the REML estimator and Hartung–Knapp adjustment. Table S10: Sensitivity analysis of adjusted hazard ratios using the REML estimator and Hartung–Knapp adjustment for primary outcomes.

**Supporting Information 2** Data S1: PRISMA 2020 checklist.

## Data Availability

Data sharing is not applicable to this article as no datasets were generated or analysed during the current study.

## References

[1] Praz F. , Borger M. A. , Lanz J. , Marin-Cuartas M. , Abreu A. , Adamo M. , Ajmone Marsan N. , Barili F. , Bonaros N. , Cosyns B. , de Paulis R. , Gamra H. , Jahangiri M. , Jeppsson A. , Klautz R. J. M. , Mores B. , Pérez-David E. , Pöss J. , Prendergast B. D. , Rocca B. , Rossello X. , Suzuki M. , Thiele H. , Tribouilloy C. M. , Wojakowski W. , ESC/EACTS Scientific Document Group , Vahanian A. , Mestres C. A. , Abid L. , Aktaa S. , Akowuah E. F. , Arbelo E. , Asselbergs F. W. , Barbato E. , Boriani G. , Brida M. , Buccheri S. , Byrne R. A. , Chioncel O. , Conradi L. , de Bonis M. , Delgado V. , Franzone A. , Haugaa K. H. , Heidecker B. , Ibanez B. , Iung B. , James S. , Køber L. , Koskinas K. C. , Landmesser U. , Lip G. Y. H. , McEvoy J. W. , Meltzer G. , Messika-Zeitoun D. , Mihaylova B. , Mindham R. , Moelgaard I. , Nielsen J. C. , Owens G. , Pasquet A. A. , Pilgrim T. , Prescott E. , Quintana E. , Rudolph V. , Sadaba R. , Sannino A. , Tanner F. C. , Urena M. , Vaartjes I. , Vrints C. , Wahba A. , Walther T. , Witkowski A. , Zeppenfeld K. , Shuka N. , Kichou B. , Chilingaryan A. L. , and Bartko P. E. , 2025 ESC/EACTS Guidelines for the Management of Valvular Heart Disease, European Heart Journal. (2025) 46, no. 44, 4635–4736, 10.1093/eurheartj/ehaf194. 40878295

[2] Blankenberg S. , Seiffert M. , Vonthein R. , Baumgartner H. , Bleiziffer S. , Borger M. A. , Choi Y. H. , Clemmensen P. , Cremer J. , Czerny M. , Diercks N. , Eitel I. , Ensminger S. , Frank D. , Frey N. , Hagendorff A. , Hagl C. , Hamm C. , Kappert U. , Karck M. , Kim W. K. , König I. R. , Krane M. , Landmesser U. , Linke A. , Maier L. S. , Massberg S. , Neumann F. J. , Reichenspurner H. , Rudolph T. K. , Schmid C. , Thiele H. , Twerenbold R. , Walther T. , Westermann D. , Xhepa E. , Ziegler A. , and Falk V. , Transcatheter or Surgical Treatment of Aortic-Valve Stenosis, New England Journal of Medicine. (2024) 390, no. 17, 1572–1583, 10.1056/NEJMoa2400685. 38588025

[3] Mori M. , Gupta A. , Wang Y. , Vahl T. , Nazif T. , Kirtane A. J. , George I. , Yong C. M. , Onuma O. , Kodali S. , Geirsson A. , Leon M. B. , and Krumholz H. M. , Trends in Transcatheter and Surgical Aortic Valve Replacement Among Older Adults in the United States, Journal of the American College of Cardiology. (2021) 78, no. 22, 2161–2172, 10.1016/j.jacc.2021.09.855. 34823659

[4] Iung B. and Vahanian A. , Epidemiology of Acquired Valvular Heart Disease, Canadian Journal of Cardiology. (2014) 30, 962–970, 10.1016/j.cjca.2014.03.022. 24986049

[5] Otto C. M. , Heartbeat: Clinical Practice Patterns for Valve Intervention in Adults With Aortic Stenosis, Heart. (2023) 109, 495–497, 10.1136/heartjnl-2023-322571. 36898730

[6] Freitas-Ferraz A. B. , Tirado-Conte G. , Dagenais F. , Ruel M. , Al-Atassi T. , Dumont E. , Mohammadi S. , Bernier M. , Pibarot P. , and Rodés-Cabau J. , Aortic Stenosis and Small Aortic Annulus, Circulation. (2019) 139, no. 23, 2685–2702, 10.1161/CIRCULATIONAHA.118.038408. 31157994

[7] Colombo A. and Leone P. P. , Adoption of TAVR in Europe vs the United States: Is it Deja-Vù?, Journal of the American College of Cardiology. (2023) 82, 1903–1905, 10.1016/j.jacc.2023.09.002. 37877905

[8] Gupta T. , Kolte D. , Khera S. , Goel K. , Villablanca P. A. , Kalra A. , Abbott J. D. , Elmariah S. , Fonarow G. C. , Rihal C. S. , Garcia M. J. , Weisz G. , Bhatt D. L. , and Collaborators , The Changing Landscape of Aortic Valve Replacement in the USA, EuroIntervention. (2019) 15, no. 11, e968–e974, 10.4244/EIJ-D-19-00381, 31403460. 31403460

[9] Wiviott S. D. and Berg D. D. , SGLT2 Inhibitors Reduce Heart Failure Hospitalization and Cardiovascular Death: Clarity and Consistency, Journal of the American College of Cardiology. (2023) 81, 2388–2390, 10.1016/j.jacc.2023.04.035. 37344039

[10] Zelniker T. A. , Wiviott S. D. , Raz I. , Im K. , Goodrich E. L. , Bonaca M. P. , Mosenzon O. , Kato E. T. , Cahn A. , Furtado R. H. M. , Bhatt D. L. , Leiter L. A. , McGuire D. K. , Wilding J. P. H. , and Sabatine M. S. , SGLT2 Inhibitors for Primary and Secondary Prevention of Cardiovascular and Renal Outcomes in Type 2 Diabetes: A Systematic Review and Meta-Analysis of Cardiovascular Outcome Trials, Lancet. (2019) 393, no. 10166, 31–39, 10.1016/S0140-6736(18)32590-X, 30424892. 30424892

[11] American Diabetes Association Professional Practice Committee for Diabetes , Bajaj M. , McCoy R. G. , Balapattabi K. , Bannuru R. R. , Bellini N. J. , Bennett A. K. , Beverly E. A. , Briggs Early K. , ChallaSivaKanaka S. , Das S. R. , and Dixon D. L. , 10 Cardiovascular Disease and Risk Management: Standards of Care in Diabetes—2026, Diabetes Care. (2026) 49, S216–S245, 10.2337/dc26-S010. PMC12690187 41358899

[12] Pittampalli S. , Upadyayula S. , Mekala H. M. , and Lippmann S. , Risks vs Benefits for SGLT2 Inhibitor Medications, Federal Practitioner. (2018) 35, no. 7, 45–48, 30766374. PMC6368009 30766374

[13] Page M. J. , McKenzie J. E. , Bossuyt P. M. , Boutron I. , Hoffmann T. C. , Mulrow C. D. , Shamseer L. , Tetzlaff J. M. , Akl E. A. , Brennan S. E. , and Chou R. , The PRISMA 2020 Statement: An Updated Guideline for Reporting systematic reviews, BMJ. (2021) 372, n71, 10.1136/bmj.n71, 33782057. PMC8005924 33782057

[14] Cochrane Handbook for Systematic Reviews of Interventions | Cochrane n.d. https://www.cochrane.org/authors/handbooks-and-manuals/handbook, (accessed May 17, 2026).

[15] Ouzzani M. , Hammady H. , Fedorowicz Z. , and Elmagarmid A. , Rayyan—A Web and Mobile App for Systematic Reviews, Systematic Reviews. (2016) 5, 10.1186/s13643-016-0384-4. PMC5139140 27919275

[16] Sterne J. A. C. , Savović J. , Page M. J. , Elbers R. G. , Blencowe N. S. , Boutron I. , Cates C. J. , Cheng H. Y. , Corbett M. S. , Eldridge S. M. , Emberson J. R. , Hernán M. A. , Hopewell S. , Hróbjartsson A. , Junqueira D. R. , Jüni P. , Kirkham J. J. , Lasserson T. , Li T. , McAleenan A. , Reeves B. C. , Shepperd S. , Shrier I. , Stewart L. A. , Tilling K. , White I. R. , Whiting P. F. , and Higgins J. P. T. , RoB 2: A Revised Tool for Assessing Risk of Bias in Randomised Trials, BMJ. (2019) 366, l4898, 10.1136/bmj.l4898. 31462531

[17] Wells G. , Shea B. , O’Connell D. , Peterson J. , Welch V. , Losos M. , and Tugwell P. , The Newcastle–Ottawa Scale (NOS) for Assessing the Quality of Non-Randomized Studies in Meta-Analysis, 2000, ReaseachGate.

[18] GRADE Handbook n.d. https://gdt.gradepro.org/app/handbook/handbook.html#h.g2dqzi9je57e, (accessed May 17, 2026).

[19] Brozek J. L. , Canelo-Aybar C. , Akl E. A. , Bowen J. M. , Bucher J. , Chiu W. A. , Cronin M. , Djulbegovic B. , Falavigna M. , Guyatt G. H. , Gordon A. A. , Hilton Boon M. , Hutubessy R. C. W. , Joore M. A. , Katikireddi V. , LaKind J. , Langendam M. , Manja V. , Magnuson K. , Mathioudakis A. G. , Meerpohl J. , Mertz D. , Mezencev R. , Morgan R. , Morgano G. P. , Mustafa R. , O′Flaherty M. , Patlewicz G. , Riva J. J. , Posso M. , Rooney A. , Schlosser P. M. , Schwartz L. , Shemilt I. , Tarride J. E. , Thayer K. A. , Tsaioun K. , Vale L. , Wambaugh J. , Wignall J. , Williams A. , Xie F. , Zhang Y. , Schünemann H. J. , and GRADE Working Group , GRADE Guidelines 30: The GRADE Approach to Assessing the Certainty of Modeled Evidence—An Overview in the Context of Health Decision-Making, Journal Of Clinical Epidemiology. (2021) 129, 138–150, 10.1016/j.jclinepi.2020.09.018, 32980429. PMC8514123 32980429

[20] Software | Cochrane n.d. https://www.cochrane.org/authors/handbooks-and-manuals/style-manual/references/reference-types/software#revman, (accessed May 17, 2026).

[21] R: The R Project for Statistical Computing n.d. https://www.r-project.org/, (accessed July 10, 2026).

[22] Paolisso P. , Belmonte M. , Gallinoro E. , Scarsini R. , Bergamaschi L. , Portolan L. , Armillotta M. , Esposito G. , Moscarella E. , Benfari G. , Montalto C. , Shumkova M. , de Oliveira E. K. , Angeli F. , Orzalkiewicz M. , Fabroni M. , Baydaroglu N. , Munafò A. R. , D’Atri D. O. , Casenghi M. , Scisciola L. , Barbieri M. , Marfella R. , Gragnano F. , Conte E. , Pellegrini D. , Ielasi A. , Andreini D. , Penicka M. , Oreglia J. A. , Calabrò P. , Bartorelli A. , Pizzi C. , Palmerini T. , Vanderheyden M. , Saia F. , Ribichini F. , and Barbato E. , SGLT2-Inhibitors in Diabetic Patients With Severe Aortic Stenosis and Cardiac Damage Undergoing Transcatheter Aortic Valve Implantation (TAVI), Cardiovascular Diabetology. (2024) 23, no. 1, 10.1186/s12933-024-02504-8, 39574095. PMC11583434 39574095

[23] Keskin B. , Hakgor A. , Khidolda Y. , Dursun A. , Akhundova A. , Savur U. , Olgun F. E. , onder O. , Gul Y. G. , Cakal B. , Demir B. , Gunes H. M. , Karaca I. O. , Guler E. , and Boztosun B. , SGLT2 Inhibitor Therapy and Long-Term Outcomes After Transcatheter Aortic Valve Implantation in Patients with Low Ejection Fraction, Medicina. (2026) 62, no. 3, 10.3390/medicina62030535, 41901616. PMC13027847 41901616

[24] Fan Y. , Chen F. , Zhao Y. , and Sun Y. , Short-Term Effects of Dapagliflozin on Non-Diabetic Patients With Severe Aortic Stenosis After Transcatheter Aortic Valve Replacement, Journal of Xinxiang Medical University. (2024) 2, 10.7683/xxyxyxb.2024.02.006.

[25] DerSimonian R. and Laird N. , Meta-Analysis in Clinical Trials, Controlled Clinical Trials. (1986) 7, 177–188, 10.1016/0197-2456(86)90046-2. 3802833

[26] Egger M. , Davey Smith G. , Schneider M. , and Minder C. , Bias in Meta-Analysis Detected by a Simple Graphical Test, BMJ. (1997) 315, 629–634, 10.1136/bmj.315.7109.629. PMC2127453 9310563

[27] Yasmin F. , Hamza M. , Fahim M. A. A. , Asghar M. S. , Ullah W. , and Alraies M. C. , Sodium Glucose Co-Transporter 2 Inhibitors in Patients With Heart Failure and Transcatheter Aortic Valve Replacement, JACC Advances. (2026) 5, 102462, 10.1016/j.jacadv.2025.102462. PMC12856177 41548478

[28] Raposeiras-Roubin S. , Amat-Santos I. J. , Rossello X. , González Ferreiro R. , González Bermúdez I. , Lopez Otero D. , Nombela-Franco L. , Gheorghe L. , Diez J. L. , Baladrón Zorita C. , Baz J. A. , Muñoz García A. J. , Vilalta V. , Ojeda-Pineda S. , de la Torre Hernández J. M. , Cordoba Soriano J. G. , Regueiro A. , Bordes Siscar P. , Salgado Fernández J. , Garcia del Blanco B. , Martín-Reyes R. , Romaguera R. , Moris C. , García Blas S. , Franco-Peláez J. A. , Cruz-González I. , Arzamendi D. , Romero Rodríguez N. , Díez-del Hoyo F. , Camacho Freire S. , Bosa Ojeda F. , Astorga Burgo J. C. , Molina Navarro E. , Caballero Borrego J. , Ruiz Quevedo V. , Sánchez-Recalde Á. , Peral Disdier V. , Alegría-Barrero E. , Torres-Llergo J. , Feltes G. , Fernández Díaz J. A. , Cuellas C. , Jiménez Britez G. , Sánchez-Rubio Lezcano J. , Barreiro-Pardal C. , Núñez-Gil I. , Abu-Assi E. , Iñiguez-Romo A. , Fuster V. , Ibáñez B. , and DapaTAVI Investigators , Dapagliflozin in Patients Undergoing Transcatheter Aortic-Valve Implantation, New England Journal of Medicine. (2025) 392, no. 14, 1396–1405, 10.1056/NEJMoa2500366, 40162639. 40162639

[29] McDonagh T. A. , Metra M. , Adamo M. , Gardner R. S. , Baumbach A. , Böhm M. , Burri H. , Butler J. , Čelutkienė J. , Chioncel O. , Cleland J. G. F. , Coats A. J. S. , Crespo-Leiro M. G. , Farmakis D. , Gilard M. , Heymans S. , Hoes A. W. , Jaarsma T. , Jankowska E. A. , Lainscak M. , Lam C. S. P. , Lyon A. R. , McMurray J. J. V. , Mebazaa A. , Mindham R. , Muneretto C. , Francesco Piepoli M. , Price S. , Rosano G. M. C. , Ruschitzka F. , Kathrine Skibelund A. , ESC Scientific Document Group , de Boer R. A. , Christian Schulze P. , Abdelhamid M. , Aboyans V. , Adamopoulos S. , Anker S. D. , Arbelo E. , Asteggiano R. , Bauersachs J. , Bayes-Genis A. , Borger M. A. , Budts W. , Cikes M. , Damman K. , Delgado V. , Dendale P. , Dilaveris P. , Drexel H. , Ezekowitz J. , Falk V. , Fauchier L. , Filippatos G. , Fraser A. , Frey N. , Gale C. P. , Gustafsson F. , Harris J. , Iung B. , Janssens S. , Jessup M. , Konradi A. , Kotecha D. , Lambrinou E. , Lancellotti P. , Landmesser U. , Leclercq C. , Lewis B. S. , Leyva F. , Linhart A. , Løchen M. L. , Lund L. H. , Mancini D. , Masip J. , Milicic D. , Mueller C. , Nef H. , Nielsen J. C. , Neubeck L. , Noutsias M. , Petersen S. E. , Sonia Petronio A. , Ponikowski P. , Prescott E. , Rakisheva A. , Richter D. J. , Schlyakhto E. , Seferovic P. , Senni M. , Sitges M. , Sousa-Uva M. , Tocchetti C. G. , Touyz R. M. , Tschoepe C. , Waltenberger J. , Adamo M. , Baumbach A. , Böhm M. , Burri H. , Čelutkienė J. , Chioncel O. , Cleland J. G. F. , Coats A. J. S. , Crespo-Leiro M. G. , Farmakis D. , Gardner R. S. , Gilard M. , Heymans S. , Hoes A. W. , Jaarsma T. , Jankowska E. A. , Lainscak M. , Lam C. S. P. , Lyon A. R. , McMurray J. J. V. , Mebazaa A. , Mindham R. , Muneretto C. , Piepoli M. F. , Price S. , Rosano G. M. C. , Ruschitzka F. , and Skibelund A. K. , 2021 ESC Guidelines for the Diagnosis and Treatment of Acute and Chronic Heart Failure, European Heart Journal. (2021) 42, no. 36, 3599–3726, 10.1093/eurheartj/ehab368.

[30] Heidenreich P. A. , Bozkurt B. , Aguilar D. , Allen L. A. , Byun J. J. , Colvin M. M. , Deswal A. , Drazner M. H. , Dunlay S. M. , Evers L. R. , Fang J. C. , Fedson S. E. , Fonarow G. C. , Hayek S. S. , Hernandez A. F. , Khazanie P. , Kittleson M. M. , Lee C. S. , Link M. S. , Milano C. A. , Nnacheta L. C. , Sandhu A. T. , Stevenson L. W. , Vardeny O. , Vest A. R. , and Yancy C. W. , 2022 AHA/ACC/HFSA Guideline for the Management of Heart Failure: A Report of the American College of Cardiology/American Heart Association Joint Committee on Clinical Practice Guidelines, Circulation. (2022) 145, no. 18, 10.1161/CIR.0000000000001063. 35363499

[31] Morel O. , Granier A. , Lochon L. , Trimaille A. , Bisson A. , Marchandot B. , Bernard A. , and Fauchier L. , Association of SGLT2 Inhibitors With Mortality and Bioprosthesis Valve Failure After TAVR: A Propensity-Matched Cohort Study, Clinical Medicine. (2025) 14, no. 19, 10.3390/jcm14197001, 41096081. PMC12524833 41096081

[32] Obeidat O. , Alayyat A. , Naser A. , Ghanem F. , Jabri A. , Brankovic M. , Jiao T. , Ruzieh M. , Haddad A. , Alexy T. , Villablanca P. , and Alhuneafat L. , Impact of SGLT2 Inhibitors on Long-Term Outcomes in TAVI Patients With Heart Failure: A Propensity-Matched Analysis, Revista Espanola de Cardiologia. (2026) 79, no. 6, 498–507, 10.1016/j.rec.2025.10.016, 41232829. 41232829

[33] Daniyal S. M. , Khatoon N. M. , Rasool A. , Gul I. , Tabish S. , Asifa S. , Khalid A. , Aftab Z. , Burhan M. , Fahim S. L. , Gondal H. T. , Nadir M. A. , Ahmed S. Z. , Ashraf D. A. , and Ahmed S. , Efficacy of SGLT2 Inhibitors on Clinical Outcomes After Transcatheter Aortic Valve Replacement: A Systematic Review and Meta-Analysis, Endocrinology, Diabetes & Metabolism. (2026) 9, no. 2, e70184, 10.1002/edm2.70184, 41746781. PMC12942055 41746781

[34] Vaduganathan M. , Docherty K. F. , Claggett B. L. , Jhund P. S. , de Boer R. A. , Hernandez A. F. , Inzucchi S. E. , Kosiborod M. N. , Lam C. S. P. , Martinez F. , Shah S. J. , Desai A. S. , McMurray J. J. V. , and Solomon S. D. , SGLT-2 Inhibitors in Patients With Heart Failure: A Comprehensive Meta-Analysis of Five Randomised Controlled Trials, Lancet. (2022) 400, no. 10354, 757–767, 10.1016/S0140-6736(22)01429-5, 36041474. 36041474

[35] Fukuta H. , Hagiwara H. , and Kamiya T. , Sodium-Glucose Cotransporter 2 Inhibitors in Heart Failure With Preserved Ejection Fraction: A Meta-Analysis of Randomized Controlled Trials, IJC Heart & Vasculature. (2022) 42, 101103, 10.1016/j.ijcha.2022.101103. PMC9399288 36032269

[36] Patel S. M. , Kang Y. M. , Im K. , Neuen B. L. , Anker S. D. , Bhatt D. L. , Butler J. , Cherney D. Z. I. , Claggett B. L. , Fletcher R. A. , Herrington W. G. , Inzucchi S. E. , Jardine M. J. , Mahaffey K. W. , McGuire D. K. , McMurray J. J. V. , Neal B. , Packer M. , Perkovic V. , Solomon S. D. , Staplin N. , Vaduganathan M. , Wanner C. , Wheeler D. C. , Zannad F. , Zhao Y. , Heerspink H. J. L. , Sabatine M. S. , and Wiviott S. D. , Sodium-Glucose Cotransporter-2 Inhibitors and Major Adverse Cardiovascular Outcomes: A SMART-C Collaborative Meta-Analysis, Circulation. (2024) 149, no. 23, 1789–1801, 10.1161/CIRCULATIONAHA.124.069568, 38583093. PMC11149938 38583093

[37] Jacquemyn X. , Hasan I. , Ogami T. , Serna-Gallegos D. , Bonatti J. , Abdullah M. , Chu D. , Kaczorowski D. J. , and Sultan I. , Sodium-Glucose Cotransporter 2 Inhibitor Use and Outcomes After Surgical Aortic Valve Replacement, Journal of Thoracic and Cardiovascular Surgery. (2026) 172, no. 3, 600–607, 10.1016/j.jtcvs.2026.02.037, 41831709. 41831709

[38] Karakasis P. , Theofilis P. , Patoulias D. , Vlachakis P. K. , Pamporis K. , Sagris M. , Ktenopoulos N. , Kassimis G. , Antoniadis A. P. , and Fragakis N. , Sodium-Glucose Cotransporter 2 Inhibitors in Aortic Stenosis: Toward a Comprehensive Cardiometabolic Approach, International Journal of Molecular Sciences. (2025) 26, no. 10, 10.3390/ijms26104494, 40429640. PMC12111810 40429640

