# Supplementary material for: Association of Sodium–Glucose Cotransporter 2 Inhibitor Use With Outcomes in Patients Undergoing Transcatheter Aortic Valve Implantation: A Systematic Review and GRADE‐Assessed Meta‐Analysis
Source: Cardiovasc Ther. 2026 Oct 5;2026:8933859. doi: 10.1155/cdr/8933859 (PMC13639379; doi:10.1155/cdr/8933859)
Supplement: Supplementary file 2 [file CDR-2026-8933859-s002.docx]

**Supplement to**: ***Association of Sodium–Glucose Cotransporter 2 Inhibitor Use With Outcomes in Patients Undergoing Transcatheter Aortic Valve Implantation: A Systematic Review and GRADE-Assessed Meta-Analysis***

**Table of contents**

| **Section** | **Title** | **Page** |
| --- | --- | --- |
| Supplementary Figure S1 | Traffic light plot of risk of bias assessment using the Cochrane Risk of Bias 2 tool for randomized controlled trials | 3 |
| Supplementary Figure S2 | Forest plot of subgroup analysis by study design for all-cause mortality in the overall population | 4 |
| Supplementary Figure S3 | Forest plot of pooled adjusted hazard ratios for all-cause mortality | 5 |
| Supplementary Figure S4 | Forest plot of all-cause mortality of the overall population at 6 months follow-up | 6 |
| Supplementary Figure S5 | Forest plot of all-cause mortality of the overall population at 12 months follow-up | ***7*** |
| Supplementary Figure S6 | Forest plot of subgroup analysis by study design for HF hospitalization in the overall population | 8 |
| Supplementary Figure S7 | Forest plot of pooled adjusted hazard ratios for HF hospitalization | 9 |
| Supplementary Figure S8 | Forest plot of HF hospitalization of the overall population at 12 months follow-up | 10 |
| Supplementary Figure S9 | Forest plot of subgroup analysis by study design for MACE in the overall population | 11 |
| Supplementary Figure S10 | Forest plot of MACE of the overall population at 6 months follow-up | 12 |
| Supplementary Figure S11 | Forest plot of MACE of the overall population at 12 months follow-up | 13 |
| Supplementary Figure S12 | Forest plot of MACE with low EF population | 14 |
| Supplementary Figure S13 | Forest plot of AKI with low EF population | 15 |
| Supplementary Figure S14 | Forest plot of LVEF of the overall population at 6 months follow-up | 16 |
| Supplementary Figure S15 | Forest plot of LVEF with LVEF <50% population | 17 |
| Supplementary Figure S16 | Sensitivity analysis forest plot of all-cause mortality of the overall population | 18 |
| Supplementary Figure S17 | Sensitivity analysis forest plot of all-cause mortality of the overall population at 12 months | 19 |
| Supplementary Figure S18 | Sensitivity analysis forest plot of all-cause mortality with low EF population | 20 |
| Supplementary Figure S19 | Sensitivity analysis forest plot of HF hospitalization of the overall population | 21 |
| Supplementary Figure S20 | Sensitivity analysis forest plot of MACE of the overall population | 22 |
| Supplementary Figure S21 | Sensitivity analysis forest plot of MACE with low EF population | 23 |
| Supplementary Figure S22 | Sensitivity analysis forest plot of MACE of the overall population at 12 months | 24 |
| Supplementary Figure S23 | Sensitivity analysis forest plot of LVEF of the overall population | 25 |
| Supplementary Table S1 | MACE Definition (Composite Endpoint) across included studies | 26 |
| Supplementary Table S2 | Summary of the search strategy for all the databases | 27 |
| Supplementary Table S3 | Full-Text Studies Excluded Due to Potential Population Overlap and Reasons for Exclusion | 28 |
| Supplementary Table S4 | Inclusion and exclusion criteria of included studies | 29 |
| Supplementary Table S5 | Newcastle–Ottawa Scale assessment of observational studies | 30 |
| Supplementary Table S6 | GRADE certainty of evidence from observational studies in the overall TAVI population by follow-up duration (12 months and 6 months) | 31 |
| Supplementary Table S7 | Additional safety outcomes reported in the DapaTAVI trial not included in quantitative synthesis | 32 |
| Supplementary Table S8 | Sensitivity analysis using the REML estimator with Hartung–Knapp adjustment for overall analysis of all outcomes | 33 |
| Supplementary Table S9 | Sensitivity analyses of subgroup and follow-up outcomes using the REML estimator and Hartung–Knapp adjustment | 34 |
| Supplementary Table S10 | Sensitivity analysis of adjusted hazard ratios using the REML estimator and Hartung–Knapp adjustment for primary outcomes | 35 |

**Supplementary Figure S1: Traffic light plot of risk of bias assessment using the Cochrane Risk of Bias 2 tool for randomized controlled trials.**

**Supplementary Figure S2: Forest plot of subgroup analysis by study design for all-cause mortality in the overall population.**

**Supplementary Figure S3: Forest plot of pooled adjusted hazard ratios for all-cause mortality.**

**Supplementary Figure S4: Forest plot of all-cause mortality of the overall population at 6 months follow-up.**

**Supplementary Figure S5: Forest plot of all-cause mortality of the overall population at 12 months follow-up.**

**Supplementary Figure S6: Forest plot of subgroup analysis by study design for HF hospitalization in the overall population.**

**Supplementary Figure S7: Forest plot of pooled adjusted hazard ratios for HF hospitalization.**

**Supplementary Figure S8: Forest plot of HF hospitalization of the overall population at 12 months follow-up.**

**Supplementary Figure S9: Forest plot of subgroup analysis by study design for MACE in the overall population.**

**Supplementary Figure S10: Forest plot of MACE of the overall population at 6 months follow-up.**

**Supplementary Figure S11: Forest plot of MACE of the overall population at 12 months follow-up.**

**Supplementary Figure S12: Forest plot of MACE with low EF population.**

**Supplementary Figure S13: Forest plot of AKI with low EF population.**

**Supplementary Figure S14: Forest plot of LVEF of the overall population at 6 months follow-up.**

**Supplementary Figure S15: Forest plot of LVEF with LVEF <50% population.**

**Supplementary Figure S16: Sensitivity analysis forest plot of all-cause mortality of the overall population.**

**Supplementary Figure S17: Sensitivity analysis forest plot of all-cause mortality of the overall population at 12 months.**

**Supplementary Figure S18: Sensitivity analysis forest plot of all-cause mortality with low EF population.**

**Supplementary Figure S19: Sensitivity analysis forest plot of HF hospitalization of the overall population.**

**Supplementary Figure S20: Sensitivity analysis forest plot of MACE of the overall population.**

**Supplementary Figure S21: Sensitivity analysis forest plot of MACE with low EF population.**

**Supplementary Figure S22: Sensitivity analysis forest plot of MACE of the overall population at 12 months.**

**Supplementary Figure S23: Sensitivity analysis forest plot of LVEF of the overall population.**

**Supplementary Table S1: MACE Definition (Composite Endpoint) across included studies.**

| **Study** | **MACE Definition (Composite Endpoint)** |
| --- | --- |
| Keskin et al. 2026 | Composite of all‑cause mortality + HF hospitalization requiring intravenous diuretics |
| Yasmin et al. 2026 | All‑cause mortality + acute myocardial infarction + cerebral infarction (stroke) + acute heart failure (systolic, diastolic, or combined) |
| Raposeiras-Roubin et al. 2025 | Death from any cause + worsening of HF (hospitalization for HF or urgent HF visit leading to IV diuretics) |
| Paolisso et al. 2024 | All‑cause death + hospitalization for HF |

**Supplementary Table S2:** **Summary of the search strategy for all the databases.**

| **Database** | **Search string** | **Filter** | **Results** |
| --- | --- | --- | --- |
| PubMed | ("SGLT2 Inhibitors"[Mesh] OR "sodium glucose cotransporter 2 inhibitor*" OR SGLT2i OR SGLT2 inhibitor* OR dapagliflozin OR empagliflozin OR canagliflozin OR ertugliflozin OR sotagliflozin OR ipragliflozin OR luseogliflozin OR remogliflozin) AND ("Transcatheter Aortic Valve Replacement" OR TAVR OR TAVI OR "Transcatheter Aortic Valve Implantation") | No filter | 48 |
| Scopus | TITLE-ABS-KEY  (("sglt2 inhibitor*" OR sglt2i OR dapagliflozin OR empagliflozin OR canagliflozin OR ertugliflozin OR sotagliflozin OR ipragliflozin OR luseogliflozin OR remogliflozin) AND ("transcatheter aortic valve replacement" OR "transcatheter aortic valve implantation" OR tavr OR tavi)) | No filter | 104 |
| Cochrane CENTRAL | ("sglt2 inhibitor*" OR sglt2i OR dapagliflozin OR empagliflozin OR canagliflozin OR ertugliflozin OR sotagliflozin OR ipragliflozin OR luseogliflozin OR remogliflozin) AND ("transcatheter aortic valve replacement" OR "transcatheter aortic valve implantation" OR tavr OR tavi) | No filter | 10 |
| Web of Science | TS=  (("sglt2 inhibitor*" OR sglt2i OR dapagliflozin OR empagliflozin OR canagliflozin OR ertugliflozin OR sotagliflozin OR ipragliflozin OR luseogliflozin OR remogliflozin) AND ("transcatheter aortic valve replacement" OR "transcatheter aortic valve implantation" OR tavr OR tavi)) | No filter | 53 |
| Embase | ('sodium glucose cotransporter 2 inhibitor'/exp OR 'sglt2 inhibitor*' OR sglt2i OR dapagliflozin OR empagliflozin OR canagliflozin OR ertugliflozin OR sotagliflozin OR ipragliflozin OR luseogliflozin OR remogliflozin) AND ('transcatheter aortic valve implantation'/exp OR tavr OR tavi OR 'transcatheter aortic valve replacement') | No filter | 244 |

Total: 459
After duplicate removal: 273

**Supplementary Table S3: Full-Text Studies Excluded Due to Potential Population Overlap and Reasons for Exclusion**

| **Study ID** | **Confounding adjustment** | **Suspected overlap** | **Retained study** | **Selection rationale** | **Residual overlap** |
| --- | --- | --- | --- | --- | --- |
| Morel et al., 2025 | 1:1 propensity score matching | TriNetX registry; overlapping TAVR population. Potential overlap period: uncertain; study period not reported. | Yasmin et al., 2026 | Prespecified 6- and 12-month outcomes and HF subgroup analyses | Cannot be excluded |
| Obeidat et al., 2026 | 1:1 propensity score matching + Cox regression | TriNetX registry (2015–2025); overlapping HF-TAVI population | Yasmin et al., 2026 | More complete extractable outcomes and low-LVEF subgroup analysis | Cannot be excluded |
| Paolisso et al., 2025 | Multivariable logistic regression | Multicenter registry (2021–2024); overlapping investigators and recruitment period | Paolisso et al., 2024 | Reported prespecified outcomes included in the meta-analysis | Possible |

**Supplementary Table S4: Inclusion and exclusion criteria of included studies.**

| **Study ID** | **Inclusion Criteria** | **Exclusion Criteria** |
| --- | --- | --- |
| Keskin et al. 2026 | • Patients with LVEF < 50% • Underwent transfemoral TAVI for severe aortic stenosis • Between January 2015 and September 2025 | • In-hospital mortality (n = 21) • Surgical conversion due to major structural complications (e.g., aortic dissection, device embolization, or annular rupture) (n = 3) • End-stage renal disease (eGFR < 30 mL/min/1.73 m² or chronic hemodialysis) (n = 12) • Missing data (n = 23) • Severe anemia (hemoglobin < 8 mg/dL) (n = 4) • Valve-in-valve TAVI (n = 10) • Active malignancy (n = 2) |
| Yasmin et al. 2026 | • Age ≥ 18 years • Diagnosis of congestive heart failure (CHF) • Underwent TAVR for aortic stenosis • Received either SGLT2 inhibitors OR standard of care | • History of cerebral infarction (stroke) BEFORE TAVR • Dialysis use BEFORE TAVR • Acute myocardial infarction (AMI) BEFORE TAVR • Atrial fibrillation (AF) and flutter BEFORE TAVR • Cardiac arrest BEFORE TAVR • Pulmonary edema BEFORE TAVR • Necrotizing fasciitis BEFORE TAVR • Amputation BEFORE TAVR • Cardiac pacemaker BEFORE TAVR |
| Raposeiras-Roubin et al. 2025 | • Severe AS undergoing TAVI • Previous AS-related HF episode (hospitalization or urgent visit with IV diuretic before TAVI) • PLUS ≥1 of: eGFR 25–75 mL/min/1.73m², diabetes mellitus, or LVEF ≤40% | • Contraindication to dapagliflozin • Current sulfonylurea or SGLT2i therapy • SBP < 100 mmHg or DBP < 50 mmHg • eGFR < 25 mL/min/1.73m² • Chronic cystitis or recurrent UTI (≥2/year) |
| Paolisso et al. 2024 | • Type 2 diabetes mellitus • Severe AS per guidelines • LVEF < 50% • EVCD stage 1–4 • Undergoing TAVI (Jan 2020–Sep 2023) | • eGFR < 20 mL/min/1.73m² • Active cancer • Follow-up < 6 months or unavailable • Inability to provide informed consent • Inadequate baseline echo for EVCD assessment • Incomplete discharge medication data |
| Fan et al. 2024 | • Meet AS diagnostic criteria and TAVR indications • Non-diabetic patients • Standardized outpatient medication • Complete clinical data with regular follow-up | • Severe hepatic/renal dysfunction or CrCl < 30 mL/min • Previous dapagliflozin use, allergy, or inability to adhere to long-term treatment • Malignant tumors or systemic diseases affecting results |

**Supplementary Table S5: Newcastle–Ottawa Scale assessment of observational studies.**

|  | **Selection** | | | | **Comparability** | **Outcome** | | | **Quality Score** | **Quality Level** |
| --- | --- | --- | --- | --- | --- | --- | --- | --- | --- | --- |
| **Study ID** | **Representativeness of the exposed cohort** | **Selection of the non  exposed cohort** | **Ascertainment of  exposure** | **Demonstration that the outcome of interest was not present at the start of the study** | **Comparability of cohorts on the basis of the design or analysis** | **Assessment of outcome** | **Was the follow-up long enough for outcomes to occur** | **Adequacy of follow-up up of cohorts** |  |  |
| Keskin et al.,2026 | * | * | * | * | ** | * | * | * | 9\9 | Good |
| Yasmin et al.,2025 | * | * | * | * | ** | * | * | * | 9\9 | Good |
| Paolisso et al.,2024 | * | * | * | * | ** | * | * | * | 9\9 | Good |
| Fan et al.,2024 |  | * | * | * | * | * | * | * | 7\9 | Good |

**Supplementary Table S6: GRADE certainty of evidence from observational studies in the overall TAVI population by follow-up duration (12 months and 6 months).**

| **Follow up** | **Outcomes** | **Anticipated absolute effects* (95% CI)** | | **Relative effect (95% CI)** | **№ of participants (studies)** | **Certainty of the evidence (GRADE)** |
| --- | --- | --- | --- | --- | --- | --- |
|  |  | **Risk with [Non SGLT2]** | **Risk with [SGLT2]** |  |  |  |
| 12 months | MACE | 301 per 1,000 | 289 per 1,000 (264 to 319) | RR 0.96 (0.88 to 1.06) | 4389 (2 non-randomised studies) | ⨁◯◯◯ Very low^a^ |
|  | All-cause Mortality | 117 per 1,000 | 48 per 1,000 (8 to 269) | RR 0.41 (0.07 to 2.29) | 4360 (2 non-randomised studies) | ⨁◯◯◯ Very low^a,b^ |
| 6 months | LVEF Change | The mean LVEF was 5.8% | MD 0.20% lower (1.83 lower to 1.44 higher) | — | 310 (2 observational studies) | ⨁◯◯◯ Very Low^a^ |
|  | MACE | 234 per 1,000 | 122 per 1,000 (30 to 473) | RR 0.52 (0.13 to 2.02) | 4,389 (2 observational studies) | ⨁◯◯◯ Very low^b,c^ |
|  | All-cause Mortality | 69 per 1,000 | 56 per 1,000 (44 to 70) | RR 0.81 (0.64 to 1.02) | 4,360 (2 observational studies) | ⨁◯◯◯  Very low^b^ |

*The risk in the intervention group (and its 95% confidence interval) is based on the assumed risk in the comparison group and the relative effect of the intervention (and its 95% CI).

CI (confidence interval), GRADE (Grading of Recommendations Assessment, Development and Evaluation), LVEF (left ventricular ejection fraction), MACE (major adverse cardiovascular events), MD (mean difference), RR (risk ratio).

a. Downgraded for imprecision due to the limited number of studies.

b. Downgraded for inconsistency due to very high heterogeneity.

c. Downgraded for indirectness due to it is a composite outcomes had different definitions across the included studies.

**Supplementary Table S7: Additional Safety Outcomes Reported in the DapaTAVI Trial Not Included in Quantitative Synthesis**

| **Outcome** | **Dapagliflozin (n=605)** | **Control (n=617)** | **P value** |
| --- | --- | --- | --- |
| Genital infection | 11 (1.8%) | 3 (0.5%) | 0.03 |
| Hypotension | 40 (6.6%) | 22 (3.6%) | 0.01 |
| Syncope | 22 (3.6%) | 13 (2.1%) | 0.11 |
| Major hypoglycemia | 4 (0.7%) | 8 (1.3%) | 0.26 |
| Nontraumatic amputation | 5 (0.8%) | 4 (0.6%) | 0.72 |
| Cancer | 30 (5.0%) | 22 (3.6%) | 0.23 |

**Supplementary Table S8:** **Sensitivity analysis using the REML estimator with Hartung–Knapp adjustment for overall analysis of all outcomes**

| **Outcome (REML + HK)** | **k** | **RR** | **95% CI** | **p-value** | **τ²** | **Q** | **I²** | **heterogeneity p-value** |
| --- | --- | --- | --- | --- | --- | --- | --- | --- |
| All-cause mortality | 4 | 0.6503 | [0.274; 1.542] | 0.2108 | 0.1938 | 11.53 | 74.00% | 0.0092 |
| HF Hospitalization | 3 | 0.6143 | [0.265; 1.426] | 0.1305 | 0.0296 | 2.33 | 14.30% | 0.3115 |
| MACE | 4 | 0.6603 | [0.322; 1.353] | 0.1629 | 0.1536 | 20.66 | 85.50% | 0.0001 |
| AKI | 2 | 0.4942 | [0.0001; 2650.256] | 0.4866 | 0.7674 | 5.31 | 81.20% | 0.0212 |
| UTI | 2 | 0.9599 | [0.043; 21.556] | 0.8947 | 0.104 | 7.36 | 86.40% | 0.0067 |
| **Outcome (REML + HK)** | **k** | **MD** | **95% CI** | **p-value** | **τ²** | **Q** | **I²** | **heterogeneity p-value** |
| LVEF change | 3 | 1.4988 | [−4.0897; 7.087] | 0.3678 | 4.2454 | 9.82 | 79.60% | 0.0074 |
| LVEDD change | 2 | −2.6599 | [−37.3995; 32.0798] | 0.5088 | 14.0264 | 15.18 | 93.40% | < 0.0001 |
| LVESD change | 2 | −2.4987 | [−36.8997; 31.902] | 0.5255 | 13.3724 | 10.39 | 90.40% | 0.0013 |

**Abbreviations:** AKI, acute kidney injury; HF, heart failure; HK, Hartung–Knapp; HR, hazard ratio; I², inconsistency statistic; k, number of included studies; LVEF, left ventricular ejection fraction; LVEDD, left ventricular end-diastolic diameter; LVESD, left ventricular end-systolic diameter; MACE, major adverse cardiovascular events; MD, mean difference; REML, restricted maximum-likelihood; RR, risk ratio; τ², between-study variance.

**Supplementary Table S9: Sensitivity analyses of subgroup and follow-up outcomes using the REML estimator and Hartung–Knapp adjustment.**

| **Outcome (REML + HK)** | ***k*** | **RR** | **95% CI** | **p-value** | **τ²** | **Q** | **I²** | **heterogeneity p-value** |
| --- | --- | --- | --- | --- | --- | --- | --- | --- |
| All-cause mortality (Low EF population) | 3 | 0.537 | [0.1299; 2.2197] | 0.2001 | 0.2321 | 7.74 | 74.20% | 0.0209 |
| All-cause mortality (6 Months) | 3 | 0.827 | [0.6872; 0.9959] | 0.048 | 0 | 0.32 | 0.00% | 0.8511 |
| All-cause mortality (12 Months) | 3 | 0.837 | [0.4498; 1.5590] | 0.3442 | 0 | 5.99 | 66.60% | 0.0501 |
| HF Hospitalization (Low EF population) | 2 | 0.5 | [0.0041; 61.2337] | 0.3178 | 0.076 | 1.31 | 23.90% | 0.2516 |
| HF Hospitalization (12 Months) | 2 | 0.651 | [0.0804; 5.2659] | 0.2328 | 0 | 0.92 | 0.00% | 0.3365 |
| MACE (Low EF population) | 4 | 0.683 | [0.3133; 1.4876] | 0.2167 | 0.1796 | 15.7 | 80.90% | 0.0013 |
| MACE (6 Months) | 2 | 0.519 | [0.0001; 3290.3950] | 0.5158 | 0.8069 | 5.71 | 82.50% | 0.0169 |
| MACE (12 Months) | 3 | 0.897 | [0.6304; 1.2771] | 0.3173 | 0.0117 | 3.58 | 44.20% | 0.1666 |
| AKI (Low EF population) | 2 | 0.513 | [0.0001; 5150.3175] | 0.5264 | 0.8988 | 5.98 | 83.30% | 0.0144 |
| **Outcome (REML + HK)** | ***k*** | **MD** | **95% CI** | **p-value** | **τ²** | **Q** | **I²** | **heterogeneity p-value** |
| LVEF% (6 Months) | 2 | -0.195 | [-9.6940; 9.3036] | 0.8374 | 0 | 0.81 | 0.00% | 0.3695 |

**Abbreviations:** AKI, acute kidney injury; HF, heart failure; HK, Hartung–Knapp; I², inconsistency statistic; k, number of included studies; LVEF, left ventricular ejection fraction; LVEDD, left ventricular end-diastolic diameter; LVESD, left ventricular end-systolic diameter; MACE, major adverse cardiovascular events; MD, mean difference; REML, restricted maximum-likelihood; RR, risk ratio; τ², between-study variance.


**Supplementary Table S10: Sensitivity analysis of adjusted hazard ratios using the REML estimator and Hartung–Knapp adjustment for primary outcomes.**

| **Outcome (REML + HK)** | ***k*** | **HR** | **95% CI** | **p-value** | **τ²** | **Q** | **I²** | **heterogeneity p-value** |
| --- | --- | --- | --- | --- | --- | --- | --- | --- |
| All-cause mortality | 4 | 0.791 | [0.551; 1.135] | 0.13 | 0.0107 | 4.1 | 26.90% | 0.251 |
| HF hospitalization | 3 | 0.576 | [0.192; 1.731] | 0.164 | 0.1008 | 4.76 | 58.00% | 0.093 |

**Abbreviations:** HF, heart failure; HK, Hartung–Knapp; HR, hazard ratio; I², inconsistency statistic; k, number of included studies; REML, restricted maximum-likelihood; τ², between-study variance.
